# Electrophysiologic Considerations in Adult Patients with Ebstein’s Anomaly

**DOI:** 10.3390/jpm14111113

**Published:** 2024-11-20

**Authors:** Ingrid Hsiung, Olubadewa A. Fatunde, Komandoor Srivathsan, Malini Madhavan, David S. Majdalany

**Affiliations:** 1 Department of Cardiovascular Medicine, University of Southern California, Los Angeles, CA 90007, USA; 2 Department of Cardiovascular Medicine, Mayo Clinic, Scottsdale, AZ 85259, USAsrivathsan.komandoor@mayo.edu (K.S.); 3 Department of Cardiovascular Medicine, Mayo Clinic, Rochester, MN 55905, USA; madhavan.malini@mayo.edu

**Keywords:** anomaly, cyanotic heart disease, congenital heart disease, electrophysiology

## Abstract

Ebstein’s anomaly (EA) is a rare cyanotic form of congenital heart disease (CHD) characterized by apical displacement of the tricuspid valve, with resultant hemodynamic and electrical manifestations. The severity of symptoms is determined by the degree of apical displacement and deformity and incompetence of the tricuspid valve. As a result, patients with EA can be severely symptomatic during infancy and childhood or can be incidentally discovered in the sixth or seventh decade of life. Hallmarks of Ebstein’s anomaly include progressive cyanosis, right-sided heart failure, and tachyarrhythmias, among which tachyarrhythmias (most commonly atrial, but also ventricular) are the most common presenting symptoms in Ebstein’s anomaly patients during adulthood. This review aims to provide insight into the genetic and electrophysiological (EP) basis underlying the tachyarrhythmias encountered when managing patients with EA.

## 1. Introduction

Ebstein’s anomaly is a rare form of cyanotic congenital heart disease (CHD) comprising <1% of all cases of CHD. In comparison, the most common cyanotic CHD (Tetralogy of Fallot) accounts for 10% of cases of all CHDs. The anatomical basis for EA is failure of the normal delamination of the tricuspid valve (TV) leaflets, leading to apical displacement of the functional TV annulus, with subsequent annular and right ventricular dilation.

**Figure 1:**
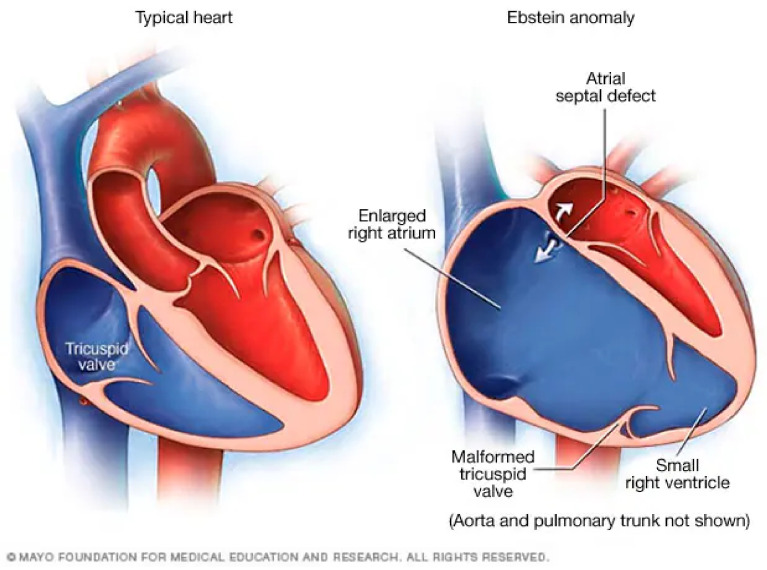
A normal anatomical heart is shown on the **left** side of the figure. Note that the tricuspid valve is slightly apical compared with the mitral valve. In a normal individual, this distance is less than 8 mm/m^2^ [1,2]. The heart pictured on the **right** of the figure is an example of a heart with an Ebstein anomaly. Note that the tricuspid valve is severely apically displaced and incompetent, resulting in severe tricuspid regurgitation and an enlarged right atrium. There is often persistent atrial communication, such as an atrial septal defect or patent foramen ovale.

Normal cardiac anatomy is compared to Ebstein’s anomaly in Figure 1. Cardiac anatomy is pictured in below.

An Ebstein anomaly (EA) is defined [3,4] by:

(1) Failure of delamination of the tricuspid valve (TV) leaflets from the underlying right ventricular (RV) myocardium, with a resultant adherence of the (septal and posterior) TV leaflets to the myocardium.
(2) Apical displacement of the functional tricuspid annular hinge points by at least 8 mm. From most to least frequent, the septal, posterior, and anterior leaflets of the TV are involved.
(3) Dilation of the “atrialized” portion of the RV with variable degrees of hypertrophy and thinning of the wall.
(4) Anterior leaflet fenestrations, redundancy, and tethering.
(5) Right atrioventricular (AV) junction (true tricuspid annulus) dilation.

There is a wide degree of anatomic variation among these defining features, with more severe forms being characterized by downward displacement of the posterior and septal leaflets being below the true annulus [3].

### 1.1. Embryology

During normal embryogenesis, the expansion of the ventricles occurs by a cycle of two processes called undermining and resorption, occurring in weeks 8–12 of embryological development. Undermining occurs when a subendocardial portion of the free wall becomes fenestrated and spongy. This process is followed by resorption of the myocardium, and this combination results in chamber enlargement. Simultaneous growth along the subepicardium preserves the ventricular wall thickness as the heart and embryo enlarges. Undermining also plays a role in formation of the RV trabeculations and TV, which occurs in two phases. The first phase is the development of the connection of the right atrioventricular junction, while the second phase involves the formation of valvar leaflets from both the ventricular musculature as well as the endocardial tissues of the AV canal and outflow segment [5]. The myocardium subtending the TV is held together by the migration of endocardial cushion tissue to the right AV junction, acting as an adhesive in order to maintain the integrity of the underlying RV [4]. Myocardial pillars—the precursors to the final TV—anchor the muscular flap to the underlying RV. Progressive thinning and fibroblastic ingrowth result in the formation of the tricuspid valve leaflets proximally and the chordae tendinae distally. These are tethered to the papillary muscles, which are spared from fibroblast ingrowth and apoptosis. The TV forms sequentially in chronological order: the anterior TV forms are the earliest, followed by the septal and posterior leaflets [3,4].

EA occurs as a result of incomplete undermining of the RV myocardium. The septal and posterior leaflets—which form late relative to the anterior leaflet—remain adherent to the myocardial wall. Therefore, the associated chordal and papillary muscle development is impaired, while the anterior leaflet becomes redundant, fenestrated, and associated with abnormal chordae tendinae [4].

### 1.2. Epidemiology and Genetics

The incidence of EA is approximately 0.005% of live births [6]. Initial studies suggested exposure to maternal lithium as a risk factor, though more recent studies note that this is not specific to EA (1.2–7.7 risk for any congenital heart disease) [3,6].

EA commonly occurs with septal defects, including secundum atrial septal defect (ASD) and/or patent foramen ovale (PFO) and pulmonary valve hypoplasia and stenosis [3,6]. Ventricular septal defect, corrected transposition of the great arteries, mitral valve prolapse, etc., are among other known associations. EA is strongly associated with the presence of accessory pathways and Wolff–Parkinson–White (WPW) syndrome, with a prevalence ranging from 10 to 52% [3,7]. There are an estimated 13–33% of EA patients with multiple accessory pathways, which are associated with a higher risk of sudden cardiac death [6,8].

The genetic etiology of EA is still under investigation. However, both deletions (10p13–p14, 1p34.3–p36.11) and point mutations (*MYH7*, transcription factor NKX2.5) have been identified [3]. The chromosome 15q has been linked to the fetal cardiac development of structures, including the tricuspid valve, and mutations may be implicated in EA. The *MYH7* gene is seen in select patients with sudden cardiac death, cardiomyopathy, and familial EA. Heterozygous mutation in NKX2.5 is implicated in multiple different CHD phenotypes: tricuspid valve anomalies, ASD, VSD, Tetralogy of Fallot, and EA [9].

Epicardium-derived cells (EPDCs) contribute to the development of both the TV valve leaflets and TV annulus fibrosus. The annulus fibrosus refers to the fibrous insulation surrounding the atrioventricular valves that function for both structural support and electrically insulating the RA from the RV, preventing conduction to the ventricles by alternative routes other than the AV node. Normally, the TV leaflet hinge points insert into the annulus fibrosus. In Ebstein’s anomaly, this, normal anatomy is altered, suggesting that abnormalities in the signaling pathways for EPDC may play a role in EA [10]. RV dilation seen in Ebstein’s anomaly further distorts the annulus fibrosus, giving rise to ventricular pre-excitation and the substrate for accessory pathways [11]. Animal models have shown an association of EPDC inhibition and the presence of accessory pathways [12]. EPDCs with particular mutations (e.g., desmoplakin deletion) gave rise to clusters of EPDCs expressing paracrine factors, including TGF-β1 and FGFs. TGF-β is involved in the development of the annulus fibrosis and has been implicated in diseases with known arrythmias involving accessory pathways, such as familial WPW syndrome. Additional research by Gaussin et al. investigated Alk3, the receptor for bone morphogenic proteins that originate from the AV myocardium, responsible for endocardial cushion formation. A knockout mouse model of Alk3 produced mice with phenotypic features of Ebstein’s anomaly, including (1) failure of leaflet delamination with (2) apical displacement of the septal leaflet and (3) distortion of the TV annulus [13].

## 2. Diagnosis

### 2.1. Early Symptoms

The confluence of abnormalities defining EA coalesce to manifest variable degrees of tricuspid regurgitation (TR) and associated heart failure, as well as rhythm abnormalities. Some EA patients, who are diagnosed in utero, do not survive infancy, while others, diagnosed on incidental imaging in the sixth and seventh decades of life, have relatively few symptoms. The age of presentation and presenting symptoms depend on co-existent cardiac structural abnormalities and arrhythmic burden [3,6].

The most common presentation of Ebstein’s anomaly for children and adolescents above age 10 years is arrhythmia [1]. Similarly, adults also present most frequently with arrhythmia (40% of all symptoms), most commonly atrial arrhythmias (e.g., atrial fibrillation/flutter, ectopic atrial tachycardia, or supraventricular tachycardia) [1]. Exercise intolerance and right heart failure symptoms (e.g., lower extremity edema, ascites, dyspnea on exertion) are less common but still frequent. Cyanosis and paradoxical emboli are possible in patients with an ASD or PFO and right-to-left shunt, which is common due to elevated RA pressure due to severe or greater tricuspid regurgitation. Asymptomatic adults with EA may simply present with an incidental cardiomegaly on a chest X-ray [3,6].

### 2.2. ECG Findings

The classic ECG findings in EA are summarized in Table 1.

Accessory pathways, including atriofascicular (Mahaim) fibers, are much more commonly seen in patients with EA compared with the general population. Manifest ventricular pre-excitation is a key feature seen in the EKG in anywhere from 5 to 25% of patients with EA [15]. Nearly half of these patients with manifest pre-excitation have multiple accessory pathways, including atriofascicular (Mahaim) pathways and multiple atrioventricular pathways (including co-existing manifest and concealed). These pathways are usually located on the right side [15,16], predominantly on the septal and posterior areas of the TV. The right-sided location of the pathways, along with the prevalent right-sided location of atriofascicular (Mahaim) fibers, suggests a link between the embryologically-based structural abnormality in the AV junction [11].

**Figure 2:**
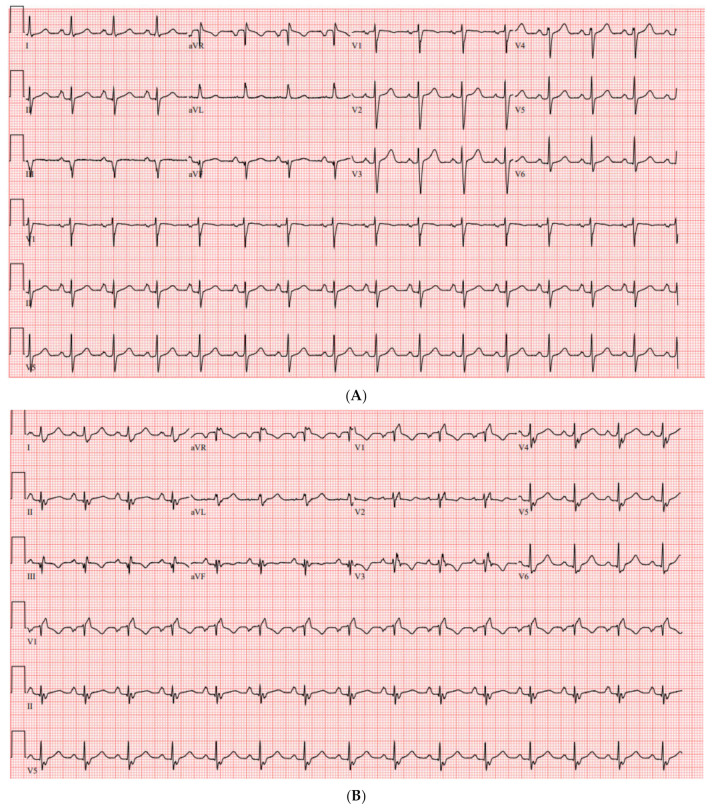
(**A**) Pre-ablation ECG, notable for right atrial enlargement and no right bundle branch block. In a patient with known EA, this is indicative of presence of a right-sided accessory pathway. There is minimal to no manifest pre-excitation. (**B**) Post-ablation ECG. Note the appearance of a right bundle branch block. There are Q waves in the inferior leads following ablation.

The EKG in 95% of Ebstein patients will show right bundle branch block (RBBB) due to the delayed activation of ventricular conduction. The absence of an RBBB pattern should alert the clinician to the presence of a right-sided accessory pathway (AP), which may carry clinical implications associated with SCD. In fact, a retrospective review found that the absence of RBBB in patients with EA carried a 98% sensitivity, 92% specificity, and 91% positive predictive value for the presence of an accessory pathway [17]. Up to one-third of patients with right-sided AP had minimal signs of ventricular pre-excitation in the EKG. A follow-up radiofrequency catheter ablation of the right-sided AP(s) revealed an underlying RBBB in 94% of patients [11,17]. This is illustrated in the pre/post-ablation ECG of a 27-year old woman with known Ebstein’s anomaly as a result of neonatal lithium exposure (Figure 2A,B).

While PR interval in patients with EA is usually short, PR interval prolongation in the EKG can also occur despite the presence of pre-excitation. This is due to intra-atrial conduction delays arising from atrialization of the RV and delayed activation time to reach the AP [11]. A prolonged PR in EA patients is due to intra-atrial conduction delay and not AV nodal conduction abnormalities as it is quite rare that these patients develop an AV block following the ablation of the pathway [17].

While tachyarrhythmias in patients with EA often involve the AP (88%), other tachyarrhythmias often manifest, including atrial fibrillation (9–22%), [11] intra-atrial reentrant tachycardias, and AV nodal reentrant tachycardia (AVNRT) (10%) [3].

The shortest pre-excited RR interval (SPERRI) between QRS complexes with delta waves should be assessed in all patients with APs, but particularly in those with EA, given their heightened risk of sudden cardiac death. A value of less than 250 milliseconds suggests a higher-risk pathway, which should be evaluated by an EP study [18].

Wide complex tachycardias are of particular interest in patients with EA, having a higher risk of SCD. The differential includes ventricular tachycardia, SVT with bystander pathway, antidromic reciprocating tachycardia, and SVT with aberrant conduction. Any patient with EA and an undefined wide complex tachycardia is a class IIa indication for an electrophysiological study.

### 2.3. Echocardiography

EA is defined by the American Society of Echocardiography (ASE) adult and pediatric guidelines as apical displacement of the tricuspid septal leaflet in the four-chamber view, either in systole or diastole, by more than ≥8 mm/m^2^ indexed [19]. Other classification systems, like Carpentier’s classification first described in 1988, stratify Ebstein’s anomaly by the atrialization of the right ventricle (RV) at varying degrees [20]. Another scale by Celermajer et al. classifies EA in neonates according to the degree of RV atrialization and area ratio of the right heart to the left heart [21]. Left ventricular function should be assessed in every echocardiogram. In patients with EA, left ventricular dysfunction is a sequelae with a heightened risk of sudden cardiac death [16].

### 2.4. Cardiac MRI

CMR imaging can be useful for EA patients with limited acoustic windows and when quantification of RV size and function are clinically necessary [2]. CMR also offers superior quantification of RV size and function compared with echocardiography. There are differing methods across institutions and not one standard method to quantify end-diastolic volume (EDV). One review suggested a pre-operative CMR-derived EDV of RV and atrialized RV (aRV) ranging from 150 to 250 mL/m^2^ [2]. Co-existing atrial (ASD and PFO) or ventricular shunt can also be quantified by CMR processing.

## 3. Associated Arrhythmias

### 3.1. Atrial Arrhythmias

Among Ebstein patients with ventricular pre-excitation, atrial arrhythmias can be fatal and can end with sudden cardiac death (SCD) [6]. In a case series of 83 patients undergoing surgery for Ebstein’s anomaly, more than half had concomitant atrial fibrillation or atrial flutter either previously documented or that were newly found upon EP testing [22].

Right atrial enlargement in EA, combined with presence of accessory pathways in the setting of a pathologic tricuspid valve, creates a complex environment where atrial tachycardia and atrial flutter can proliferate. Mapping these focal triggers and re-entry circuits prior to tricuspid valve surgery is essential; otherwise, a Cone procedure or new prosthetic valve may interfere with ability to accurately map the atrial arrhythmia. A multicenter study by Moore et al. reviewed EA patients with CTI-dependent flutter, focal atrial tachycardia, and SVT, who had catheter ablation before or after they underwent tricuspid valve surgery. The group of patients who had catheter ablation after valve surgery had significantly longer procedure times (4.3 h vs. 3.3 h, *p* = 0.003) and longer fluoroscopy times (31 min vs. 18 min, *p* = 0.001); short-term success rates were also lower in the group with ablation occurring after valve surgery (acute success 81% vs. 94%, *p* = 0.03). Lower success rates in the group with ablation after valve surgery were attributed to the lack of successful ablation of TV annular substrates, as stratifying the groups showed the lowest acute success rate to be among patients with a TV ring or replacement (73%), higher acute success rates to be among patients with TV repair that did not affect the annulus (92% success rate), and the highest acute success rates to be among patients with no valve surgery (94%, *p* = 0.01) [23].

Miguel et al. found that among 682 patients with EA, the prevalence of atrial fibrillation was 18% and the prevalence of atrial flutter/atrial tachycardia was 21%. In the same study, among EA patients without atrial arrhythmias, the 10-year cumulative incidence of developing atrial fibrillation was 16%, while developing atrial flutter/atrial tachycardia was 22%. Associated risk factors included larger left and right atrial size, age, and strain. In particular, recurrent atrial fibrillation was linked with higher levels of right atrial reservoir strain and older age [24,25].

Indeed, atrial arrhythmia recurrence appears to be common among patients with EA. A study by Hassan et al. looked at EA patients with atrial arrhythmias, with atrial arrhythmia recurrence as the primary outcome. Among 22 patients, of whom 63.5% had atrial flutter (with all but one of these patients having CTI-dependent flutter), 22.7% had focal AT, and 9.1% had atrial fibrillation, the recurrence rate at 1 year was 10.0%, while at 5 years, it was 41.2%. The study did not find echocardiographic predictors of arrhythmia recurrence [26].

### 3.2. Supraventricular Tachycardia

Pre-excitation and Wolff–Parkinson–White (WPW) syndrome are more commonly seen in patients with Ebstein’s anomaly than other congenital heart defects [27]. Approximately one-third or more of Ebstein’s patients have accessory pathways that are associated with ventricular pre-excitation [6]. In certain case series, up to 62% of patients have EKG findings consistent with pre-excitation, and in those without typical EKG features of pre-excitation, a lack of right bundle branch block paired with recurrent tachyarrhythmia was extremely predictive (98% sensitive and 92% specific) of the accessory pathway being present [17]. Up to 30% of patients with Ebstein’s anomaly have been reported to have atrioventricular reentrant tachycardia (AVRT). Accessory pathways are most often right-sided, located on the posterior portion of the tricuspid annulus, with pathways located along the atrialized right ventricle (RV) being linked with later radiofrequency ablation (RFA) failure and AVRT recurrence [7,28]. A study looking at SVT in Ebstein anomaly patients found that 52/76 patients had an isolated accessory pathway (AP), 12/76 had atrial flutter, and 3/76 had atrioventricular nodal reentrant tachycardia (AVNRT). Of the patients, 9/76 had AP plus another arrhythmia, and 40/78 were right-sided and 37 had a septal location, with only 1 being left-sided AP. When stratified accordingly to early-era procedures (performed from 1990 to 2004) and the recent era (2005–2019), acute success rates for first AP ablation were similar (89% vs. 88%, *p* = 0.48). Recurrence rates at 1 year were higher for the early era vs. the recent era (62% vs. 19%, *p* = 0.005) [29].

### 3.3. Ventricular Arrhythmia (VA)

**Figure 3:**
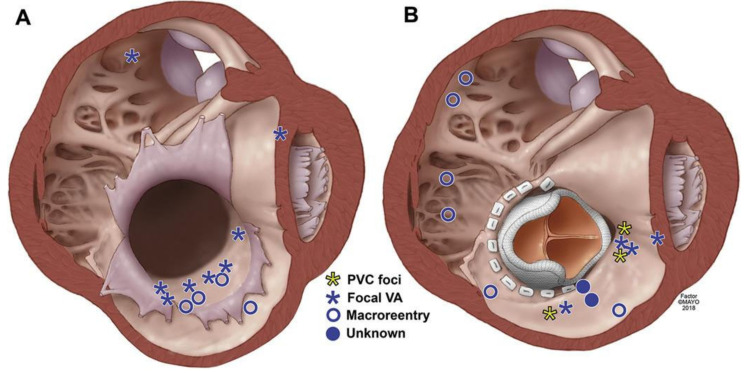
This illustration depicts the right ventricle in a typical patient with Ebstein’s anomaly prior to (**A**) and post-TV replacement (**B**). The axial section of the heart is visualized in a left anterior oblique view, from the ventricle, below the valve. The most common site of origin for ventricular arrhythmias in unoperated patients was in the atrialized right ventricle. Post TV replacement, the sites of origin (focal) and slow zones (macroreentry) of the ventricular arrhythmias were diverse [32].

The development of VA in patients with EA confers a six-fold higher risk of sudden cardiac death [7,30]. VA in patients with EA have been found to be predominantly focal or macro-reentrant. Among patients who have not undergone surgical correction, VAs often arise from the atrialized RV due to the maladaptive properties of slowed conduction velocity and shortened effective refractory period [8,31]. Conversely, those who have had prior surgical intervention have both focal VA due to an injured myocardium or Purkinje fibers as well as macro–re-entry from a post-surgical isolated scar (Figure 3) [32].

A comprehensive study by Jost et al. reviewed the risk factors associated with sudden cardiac death (SCD) in 968 EA patients. In a multivariate analysis, ventricular tachycardia was the risk factor associated with the highest adjusted hazard ratio (6.02; 95% CI 3.17, 11.44; *p* < 0.001). Other multivariate predictors of SCD included prior TV surgery (5.94; 95% CI 2.31, 15.25; *p* < 0.001), heart failure (HR 5.64; 95% CI 3.20, 9.94; *p* < 0.001), pulmonic stenosis (HR 3.42; 95% CI 1.62, 7.25; *p* = 0.001), syncope (HR 2.03; 95% CI 1.13, 3.65; *p* = 0.019), and hemoglobin > 15 g/dL (HR 2.05; 95% CI 1.09,3.87; *p* = 0.026). After TV surgery, the risk of SCD at 1 year, 5 years, 10 years, and 20 years post-operatively was 2.2%, 3.9%, 6.0%, and 10.8%, respectively. In comparison, for all EA patients, the SCD risk at age 10 years, 20 years, 50 years, and 70 years was 0.8%, 1.9%, 8.3%, and 14.6%, respectively [19]. Interestingly, there appeared to be a high incidence of inappropriate ICD therapies in EA patients. Within the same study, out of 968 patients, 24 had ICD implantations: 14 for secondary prevention (where 7 received appropriate shocks for VT and 3 received inappropriate shocks for AT) and 10 for primary prevention (where 0 received appropriate shocks and 4 received inappropriate shocks).

## 4. Indications for Intervention

### 4.1. EP Study

A catheter-based EPS and ablation is recommended in all patients with EA with evidence of pre-excitation by ECG and/or recurrent supraventricular tachycardia, undefined wide-complex tachycardia, or syncope [3,16,18].

EPS is recommended in all adult patients prior to operative intervention for the identification and potential treatment of atrial arrhythmias. If percutaneous ablation is not possible, then mapping can aid intraoperative ablation [3,16]. The 2016 expert consensus statement on use of catheter ablation in patients with congenital heart disease recommends that due to high rates of pre-operative arrhythmia, patients with Ebstein’s anomaly should undergo EP study prior to tricuspid valve surgery, even in the absence of symptoms [16,33].

### 4.2. EPS and Catheter Ablation

Having multiple pathways is likely more than a linear increase in risk. A study that examined the electrophysiological studies of patients with multiple APs versus a single AP found that those multiple APs were more likely to have a shorter pathway effective refractory period in both antegrade (multiple APs 233 ± 18 vs. single AP 270 ± 32 msec, *p* < 0.05) and retrograde 238 ± 10 vs. 262 ± 21 msec, *p* < 0.05) directions, shorter RR interval during atrial fibrillation (228 ± 16 vs. 250 ± 21 msec, *p* < 0.05), higher incidences of antidromic tachycardia (15% vs. 0.8%), and atrial fibrillation (40% vs. 12.8%, *p* < 0.05) [34].

It has long been established that the catheter ablation of multiple pathways is superior to medical therapy, as multiple pathways may have different electrophysiologic properties. Thus, antiarrhythmic drugs may have different effects on different accessory pathways, resulting in unsatisfactory control of tachycardias with antiarrhythmic drugs alone [34].

The mapping and successful catheter ablation of these pathways can be challenging for various reasons, including the enlarged right atrium and apical displacement of the TV, signals from ipsilateral adjacent pathways fusing multiple pathways not being apparent until one pathway is ablated, temporary loss of function of a pathway due to catheter trauma, and repetitive concealed conduction. Most importantly, there are unusually fractionated and low-amplitude electrograms (EGMs) at and below the AV groove, making mapping of the accessory pathway difficult [11,16,34]. Various techniques described in the literature may help overcome these challenges. Intracoronary mapping using fine electrode wires may be useful in the setting of low-amplitude EGMs [16]. A post mortem examination of 33 patients with EA found that nearly one-half of hearts had a prominent right AV ridge separating the right atrium from the atrialized portion of the RV [11,35]. This anatomic abnormality is positively correlated with the presence of an accessory pathway and may be visible in intracardiac ultrasound.

Roten et al. found that the success rate was 80% for the first catheter ablation of CTI-dependent flutter (*n* = 5) and 100% for inter-atrial reentry tachycardias (*n* = 8); the authors postulated that the success rate for CTI flutter was lower than in patients with normal anatomy due to the dysplastic tricuspid annulus in Ebstein’s patients [36].

#### 4.2.1. Cases

The following clinical cases will illustrate the principles described above.

#### 4.2.2. Case 1

**Figure 4:**
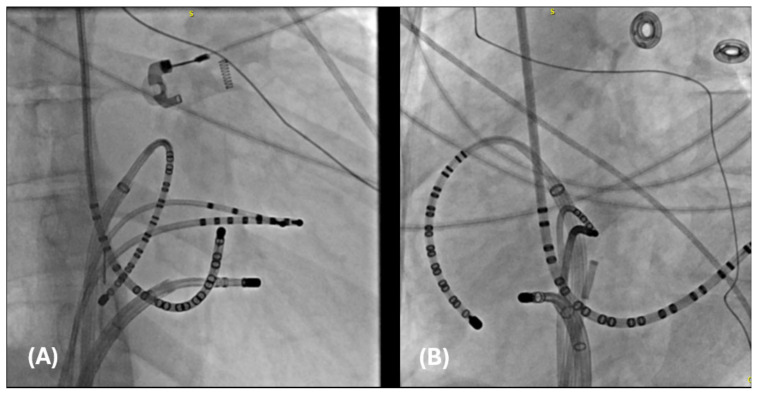
Catheter placement of catheters in preparation for an invasive electrophysiological study. (**A**) A right anterior oblique (RAO) image of a duodecapolar Cristacath catheter (A20) catheter inside an SR0 sheath along the tricuspid annulus, another duodecapolar catheter placed in the coronary sinus, a quadripolar catheter advanced to the RV apex, an octapolar catheter advanced to the His position, and an ablation catheter within a steerable sheath positioned in the anterolateral tricuspid annulus. (**B**) The same catheters are pictured in the left anterior oblique (LAO) view.

**Figure 5:**
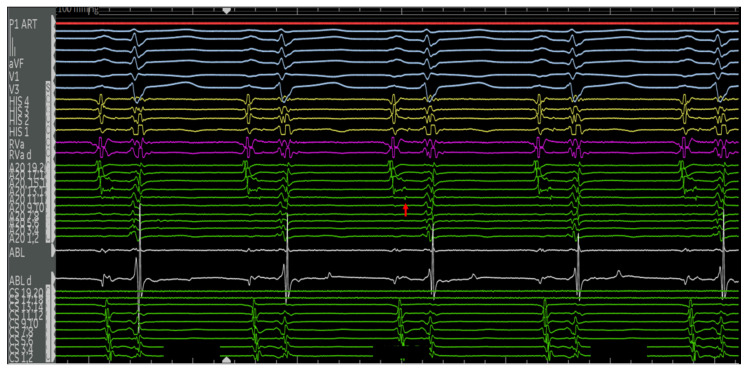
Electrograms of the aforementioned 27F with Ebstein’s anomaly in sinus rhythm. Catheters used and their respective positions are indicated in Figure 4. The surface ECG and intracardiac EGMs are labeled on the left side of the figure. Normal AH and HV intervals are present at the baseline with no manifest pre-excitation. There is a near-field potential on the A20 catheter along the lateral tricuspid annulus (red arrow). This precedes the His signal and is most consistent with a pathway potential.

**Figure 6:**
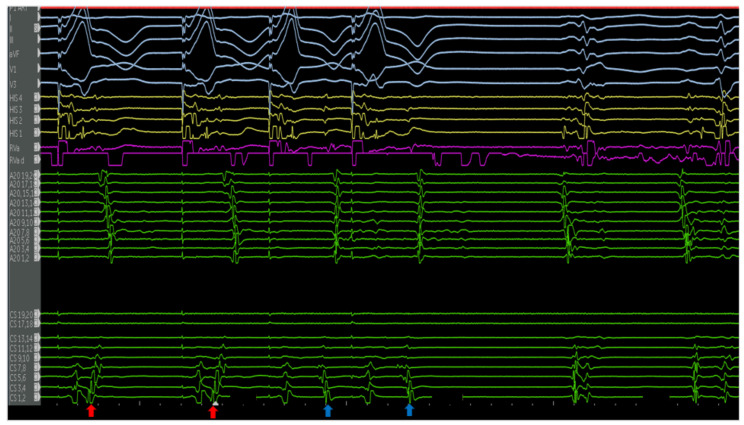
Ventricular extrastimulus testing revealed multiple retrograde atrial activations, including an earliest atrial activation in the distal CS (red arrows). This finding was reproducible, suggesting the presence of a left lateral pathway. The atrial activations of the extrastimulus beats (blue arrows) possibly represent fusion with the pathway. The VA times are stable for the first two beats (left lateral pathway) and decremental, following the vetricular extrastimuli. The a VH is stable and HA is decremental, suggestive of retrograde conduction through the AV node.

**Figure 7:**
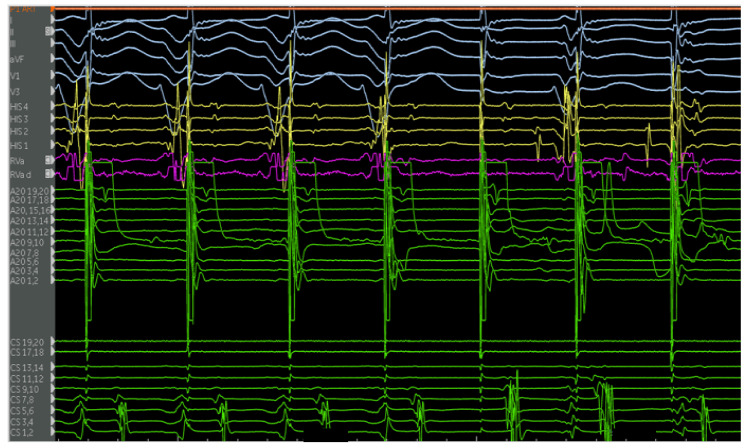
Atrial decremental pacing from the lateral tricuspid annulus demonstrates maximal pre-excitation. The end refractory period of the antegrade-conducting pathway occurs at 320 milliseconds, hallmarked by a loss of pre-excitation. Following this, the His signal decrements, indicative of antegrade conduction through the AV node.

**Figure 8:**
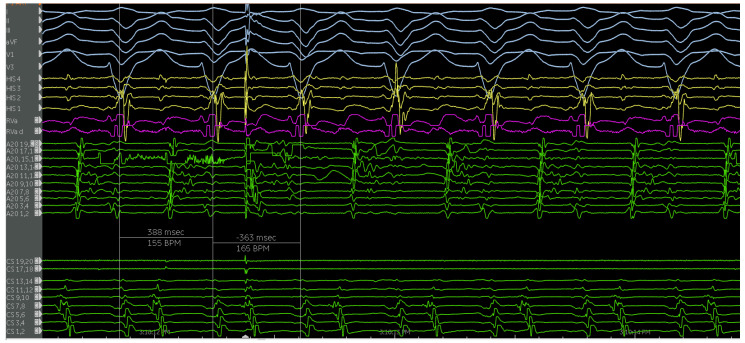
Electrograms show a wide complex tachycardia with a 1:1 V:A relationship with the earliest retrograde A occurring in the mid-CS, suggesting eccentric activation. This retrograde activation is identical to the decremental retrograde conduction seen during VEST. An early premature atrial contraction delivered from the A20 catheter in the lateral right atrium pre-excited the ventricle with an identical QRS morphology and reset the tachycardia, ruling out ventricular tachycardia. There was no advancement in the His A, septal A, ruling out nodoventricular tachycardia or AVNRT with a bystander AP. These findings confirm the participation of the pathway as the antegrade limb in this antidromic reciprocating tachycardia. The septal A was not advanced, making activation of the ventricle via a nodoventricular tract unlikely. These findings are consistent with the antegrade limb of the circuit being an atriofasicular (Mahaim) pathway. The A20 atrial electrogram suggests an oblique orientation of the pathway. Catheter positions are detailed in Figure 3.

**Figure 9:**
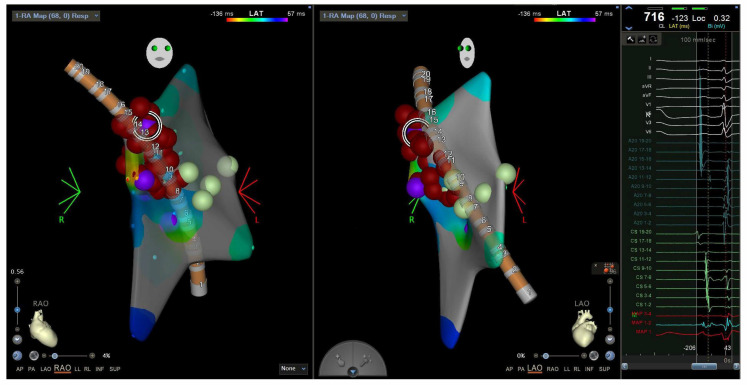
Electroanatomic map showing an RAO (**left**) and LAO (**middle**) view of the A20 catheter and radiofrequency ablation lesions in the lateral tricuspid annulus corresponding to the EGM of the pathway potential and earliest point of activation (**right**). Catheter positions were previously described in Figure 4. RAO = right anterior oblique. LAO = left anterior oblique. EGM = electrogram.

The first is a 27-year-old woman with mild EA and moderate TR (discussed earlier in Figure 2A,B). She was taken to the EP laboratory for progressively symptomatic tachycardia. The following figures show fluoroscopy images (Figure 4A,B) and electrograms (Figure 5, Figure 6, Figure 7, Figure 8 and Figure 9), the electroanatomic map, and ablation lesions. She was found to have multiple pathways—an atriofascicular (Mahaim) pathway and a retrograde left lateral pathway, which participated in ORT. Both pathways were ablated, and thus far, she has had no recurrence. Detailed descriptions of the study findings are present in the figure text.

#### 4.2.3. Case 2

**Figure 10:**
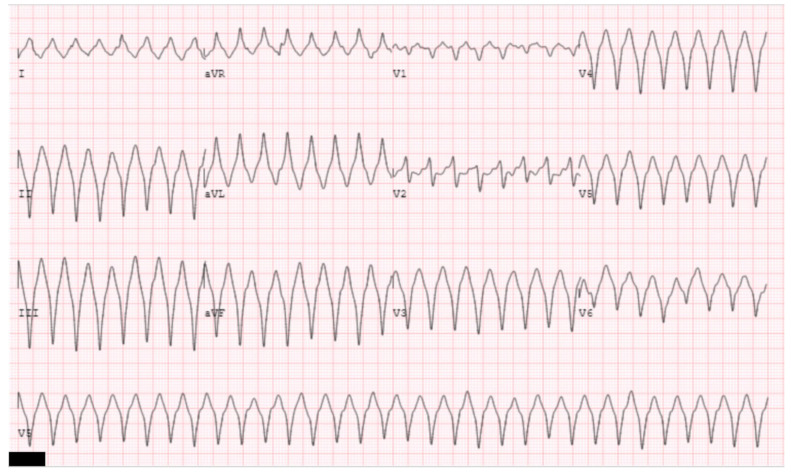
The clinical ventricular tachycardia is illustrated. ECG morphology of left bundle, left, superior axis suggests a ventricular tachycardia originating from the basal, inferior wall of the right ventricle. Note there is a reverse pattern break likely indicating exit near the basal septum [37].

**Figure 11:**
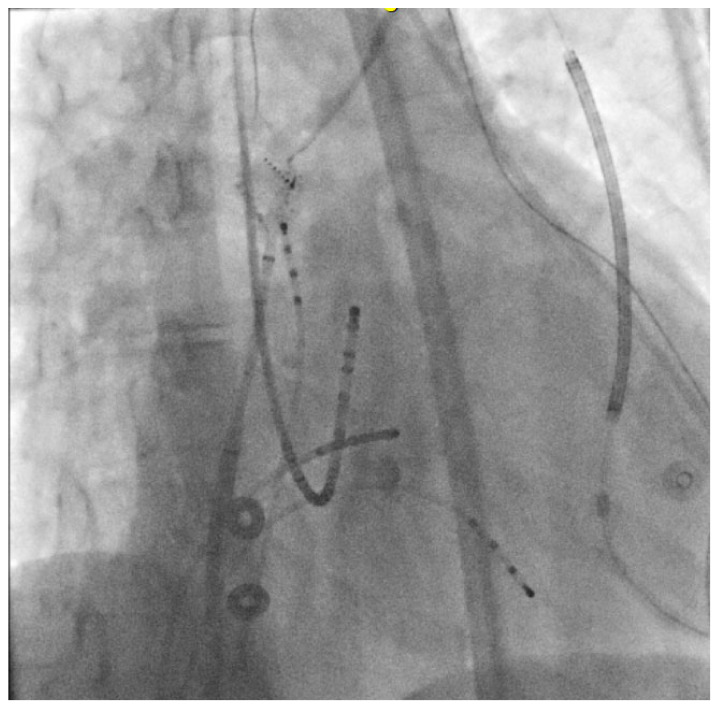
An RAO view is shown of the catheters for this electrophysiological study. There are quadripolar catheters present in the right atrium and ventricle. The His octapolar catheter and CS duodecapolar catheters are present in standard positions. A PentaRay mapping catheter present within a steerable sheath in the high right atrium. Patient’s subcutaneous ICD lead is also visualized.

**Figure 12:**
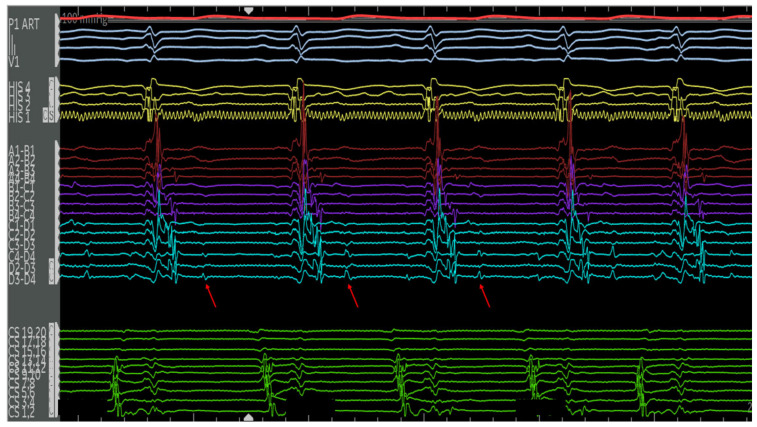
Electrograms on the PentaRay mapping catheter (Rows A1–D4) in sinus rhythm are notable for highly fractionated signal following the ventricular electrogram indicating local abnormal ventricular activity. There are also late potentials present after greater than 100 milliseconds of local delay. Selected late potentials are highlighted (red arrows). These are indicative of significant scar and poor conduction through this area. These areas were later targeted for ablation. Catheter positions are previously described in Figure 11.

**Figure 13:**
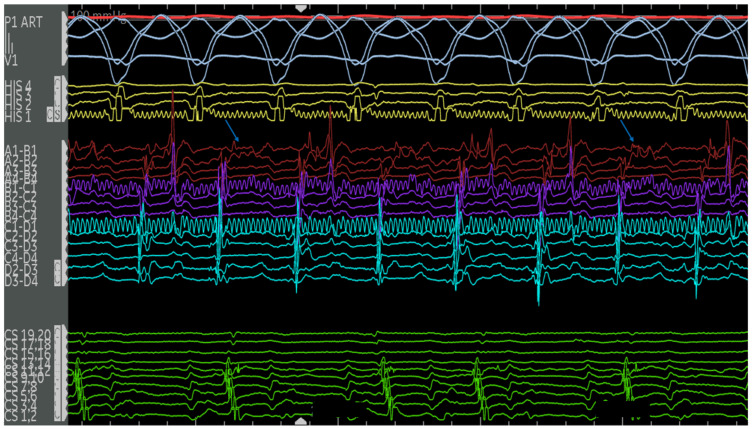
Patient is in her clinical VT (left bundle, left superior axis) at a cycle length of approximately 380 milliseconds. Note the mid-diastolic signals seen on the A20 catheter. Signals of interest (high frequency, low amplitude, highly fractionated) are present on various electrodes on the PentaRay catheter, with select signals highlighted (blue arrows). Catheter positions are previously described in Figure 11.

**Figure 14:**
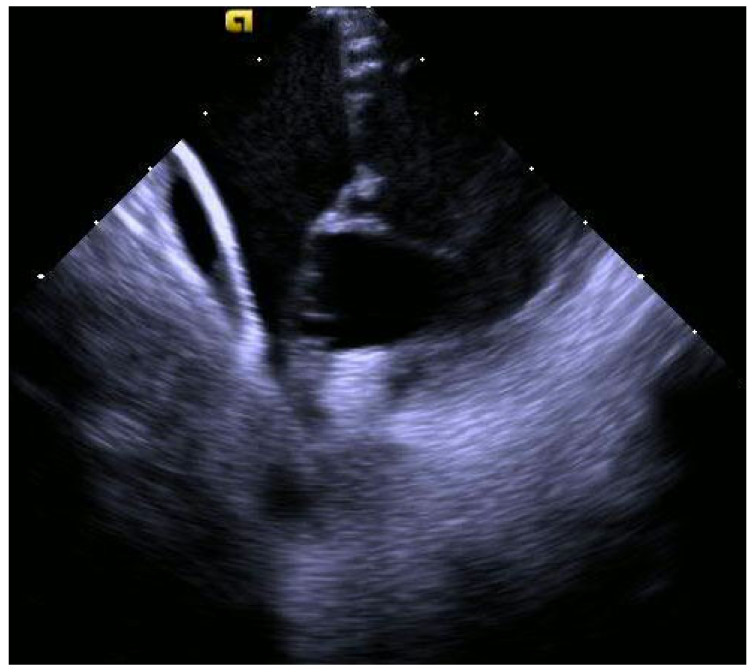
The irrigated radiofrequency ablation catheter is visualized in one of the areas of interest previously described (Figure 12 and Figure 13). Note the catheter position, atrial to the apically displaced tricuspid valve.

**Figure 15:**
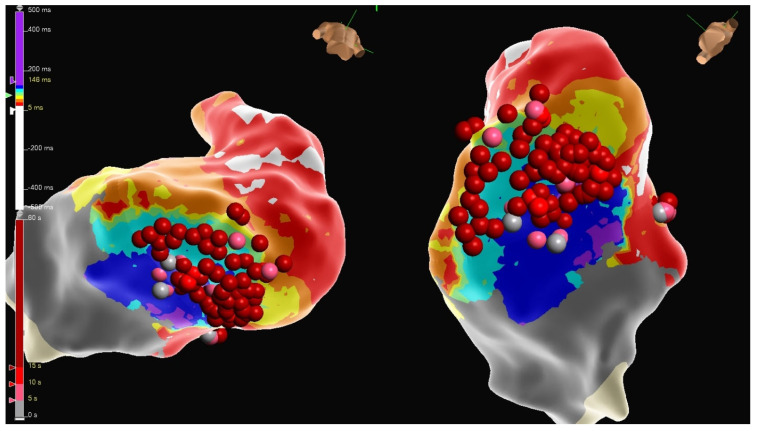
Substrate ablation was performed on the areas of interest identified in the electroanatomic mapping. A local activation timing map is displayed.

The second case is a 50-year-old woman with EA with a severely apically displaced TV (TV displacement index 19.8 mm/m^2^) on sotalol due to ventricular tachycardia (cycle length of 300 milliseconds) discovered incidentally during an unrelated hospitalization years earlier. Following multiple episodes of syncope, a subcutaneous ICD was placed. After receiving a shock, she was taken to the EP lab for the study and ablation of her ventricular tachycardia. Her clinical ventricular tachycardia is illustrated in Figure 10, Figure 11, Figure 12, Figure 13, Figure 14 and Figure 15 below. Her electrograms were notable for the highly fractionated and delayed ventricular electrograms in sinus rhythm in the inferior wall of her atrialized right ventricle. Her clinical ventricular tachycardia was similar to two of the four VTs elicited. However, hemodynamic instability limited extensive entrainment. Extensive substrate ablation was performed with radiofrequency ablation in the inferior wall of the right ventricle. There have been no known recurrences.

### 4.3. Surgical Intervention

#### Intra-Op EP Procedures

Khositeth et al. retrospectively reviewed 83 patients undergoing surgery for Ebstein’s anomaly and found that out of 48 patients with atrial fibrillation or flutter, 38 underwent right-sided surgical MAZE and 10 underwent cavo-tricuspid isthmus (CTI) cryoablation, with an overall 75% freedom from recurrence at approximately 3 years [22].

## 5. Complications Post-Intervention

Patients who have undergone surgical correction more frequently have VAs that originate from prior surgical scars (macro re-entry), injured myocardia (focal VA), or the Purkinje fibers (focal VA) [32].

There is a reported incidence of coronary artery damage in 1.7% of a pediatric population post-ablation of APs in patients with a structurally normal heart and a posteroseptal accessory pathway.

### Cardiac Implantable Electronic Device (CIED) Management

**Figure 16:**
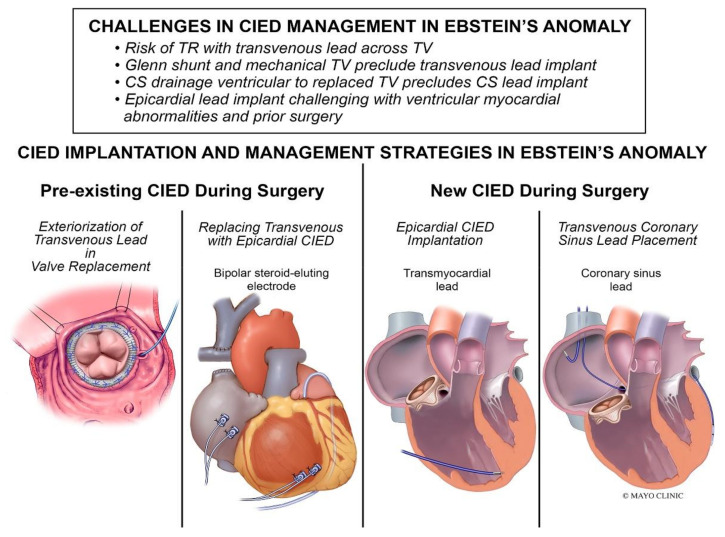
CIED implantation and management strategies in patients with EA [30].

Management of cardiac implantable electronic devices (CIEDs) can be challenging (Figure 16). A retrospective review by Tan et al. found that among 93 patients with Ebstein’s anomaly and a CIED procedure, 48% (*n* = 45) received a CIED at the time of tricuspid valve surgery, and 82% (*n* = 37) of the CIEDs implanted during surgery were epicardial systems [30]. The same study found that among patients with pre-existing transvenous RV leads at the time of tricuspid valve surgery (*n* = 20), the RV lead was externalized in 75% of patients (*n* = 15) and was extracted with the epicardial lead implantation in 25% of patients (*n* = 5) [30].

## 6. Conclusions

EA is a relatively uncommon right-sided valvular cardiomyopathy. There are a wide variety of clinical manifestations. Understanding the condition requires knowledge of embryology, pathophysiology, hemodynamics, multimodality cardiac imaging, and electrophysiology. The earliest and most common complications are tachyarrhythmias, the catheter management of which can be usually achieved with low complication rates and low rates of recurrence. More advanced attempts to address rhythm and hemodynamic issues can be pursued as needed.

## Figures and Tables

**Table 1:** **Typical ECG features in patients with EA.** Modified from the 2014 PACES/HRS consensus statement on the recognition and management of arrhythmias in adult congenital heart disease [14].

Rhythm	Normal sinus rhythm Possible ectopic atrial rhythm Supraventricular tachycardia Atrial fibrillation; intra-atrial reentrant tachycardia (40%)
PR interval	Usually long (1st degree AVB) Short with WPW
QRS axis	Normal Left axis deviation
QRS configuration	Low-amplitude multiphasic atypical right bundle branch block (RBBB) If no RBBB present, consider right sided pathway
Atrial enlargement	Common, with Himalayan p waves
Ventricular hypertrophy	None; diminutive RV
Unique features	Accessory pathway common Q waves present in inferior (II, III, avF) and anterior (V1–V4) leads
Wide complex tachyarrhythmias	Multiple APs have a higher probability of antidromic reciprocating tachycardia Ventricular tachycardias likely a late cause of SCD

## Data Availability

No new data were created or analyzed in this study.

## Abbreviations

Adult congenital heart disease	(ACHD)
Accessory pathway	(AP)
Atrial septal defect	(ASD)
Atrioventricular	(AV)
Atrioventricular nodal reentrant tachycardia	(AVNRT)
Ebstein anomaly	(EA)
Patent foramen ovale	(PFO)
Right ventricle	(RV)
Supraventricular tachycardia	(SVT)
Sudden cardiac death	(SCD)
Tricuspid valve	(TV)
Ventricular arrhythmia	(VA)
Wolff–Parkinson–White	(WPW)
